# The unexpected epidural: a case report

**DOI:** 10.1186/s12871-015-0062-4

**Published:** 2015-06-04

**Authors:** Riccardo Pinciroli, Roberto Fumagalli

**Affiliations:** 1 Department of Anesthesia and Critical Care, Anesthesia and Critical Care Service 1, Niguarda Cà Granda Hospital, Piazza dell’Ospedale Maggiore, 3, 20162 Milan, Italy; 2 Department of Health Sciences, University of Milan-Bicocca, Monza, Italy

**Keywords:** Breakage, Retained, Epidural, Catheter, Fragment, Complications

## Abstract

**Background:**

We report the peculiar case of a patient with a retained large epidural catheter fragment, incidentally found 12 years after its placement. Our primary aim is to emphasize how the breakage and retention of even exceptionally large portions of this device can go undetected. The patient can be completely asymptomatic and, with no clue that such a foreign body exists, the presentation of its potential complications can be subtle and misleading. To our knowledge, this is the first reported case of the incidental discovery of such a large fragment so many years after its placement. No consensus exists about how to handle this complication, therefore our report adds to the amount of available evidence.

**Case Presentation:**

A 53-year-old caucasian female with a history of diverticulitis requiring multiple hospitalizations underwent laparoscopic sigmoidectomy. The early postoperative period was complicated by peritonitis, demanding an urgent “second-look” exploratory laparoscopy. Nine days post-operatively, a filiform metallic object in the upper-quadrant was noted on x-ray. No epidural had been placed for either one of her recent surgeries. Given the patient’s history, the object was initially thought to be a retained surgical sponge. Previous studies, however, showed that the same image was already present preoperatively. Upon further questioning, the patient reported an epidural being placed twelve years before, at the time of her pregnancy. No mention of breakage had been made to her at that time, nor a retained foreign body was ever reported afterwards, despite her many imaging exams. She also never experienced any symptoms. A 15 cm fragment of a wire-reinforced catheter was surgically retrieved under local anesthesia and fluoroscopic guidance.

**Conclusion:**

Breakage of the epidural catheter with fragment retention is a known complication of this device, possibly leading to devastating sequelae. The fragment can go undetected for years. In this case our finding was incidental and the patient was asymptomatic. However, in the event a neurologic complication arose, the identification of the unknowingly retained epidural as the causative agent could have been difficult and delayed, with potential harm to the patient.

## Background

The epidural catheter is a widely used medical device. Anesthesiologists ordinarily place it in order to administer anesthesia and manage pain in several different clinical scenarios. Besides the risks related to the delivery of anesthetics and analgesics through the epidural route, additional hazards are those related to the placement, the permanence or the removal of the catheter itself. Although being considered a rather safe procedure, adverse events like dural punctures, spinal hematomas or epidural abscesses have been continuously reported since the introduction of this technique. The outcome of these complications can be devastating, with major neurological sequelae [1].

Among the different issues that the anesthesiologist may face while dealing with an epidural, breakage of the catheter is a rare but troublesome event [2].

Entrapment around the spinal ligaments, kinking, knotting or looping of the catheter within the epidural space or subcutaneous tissues, defective devices, and a poor handling technique by the operator are among the potential causes of fracture [3].

Here we want to report a peculiar case of a long-term retained large epidural catheter fragment that was incidentally identified and then retrieved 12 years after its placement.

## Case presentation

A 53-year-old caucasian G1P1 otherwise healthy female patient was known for a long history of diffuse right and left colonic diverticular disease, with repeated episodes of abdominal pain due to recurrent diverticulitis.

She presented to the emergency department at our institution with severe left-lower-quadrant abdominal pain, cramping and anorexia. Physical examination revealed a soft abdomen, with localized left lower quadrant sharp tenderness, guarding and moderate rebound on examination. Abdominal Computed Tomography (CT) scan showed bowel wall thickening and contrast-enhancement along a 45 mm portion of the medium-to-distal sigmoid colon. A marked increase in soft tissue density was also observed within the pericolonic fat, with evidence of a 23 mm paramedian fluid collection in the pre-sacral area. The patient was admitted to the surgical ward and antibiotic coverage was started together with analgesics. Symptoms resolved quickly, she was discharged home a week later with a diagnosis of self-limited diverticular microperforation, and an outpatient follow-up was scheduled for elective surgical planning.

Four months later, she underwent a laparoscopic sigmoidectomy with a double-stapled colo-rectal transanal anastomosis. Surgery was successful, but on post-operative day 2, the patient developed signs of peritonitis, with pain, fever, and elevated markers of inflammation. After close clinical observation, and careful re-evaluation of the case, on post-operative day-6 a second exploratory laparoscopy was performed. The procedure ruled out an anastomotic leak, as well as no abnormalities were observed at any level within the abdominal cavity. Patient improved post-operatively and was discharged home a week later.

**Fig. 1:**
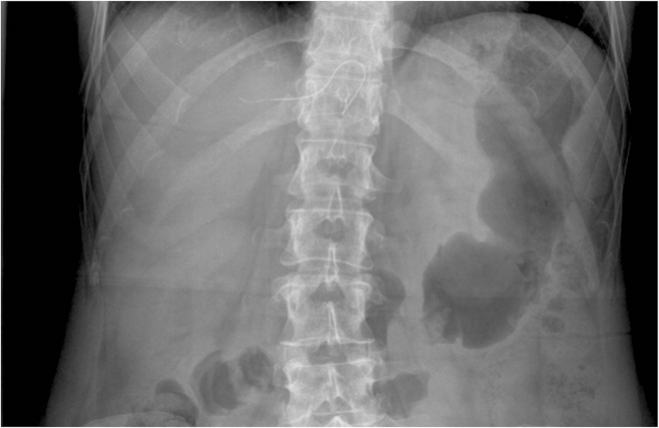
Retained epidural fragment at abdominal X-ray. A pre-operative abdominal plain X-ray showing the retained epidural fragment at T11-12 level. The identification of the catheter was repeatedly missed over the previous 12 years. Note the significant bowel distension related to the patient’s underlying disease for which the x-ray was being taken

Nine days after hospital discharge, she presented to the outpatient clinic for a scheduled follow up. A control abdominal plain X-ray was obtained, confirming the stability of her condition. However, looking at the X-ray, the staff surgeon noted a filiform metallic object projected around the midline at the upper abdominal quadrant level (T11-T12). Considering patient’s history, a retained surgical item was strongly suspected, with the wire-like image being potentially consistent with the radiopaque marker of a surgical sponge. In order to clearly establish the nature of this foreign body, at first the surgical dressing was removed, but no superficial radio-opaque material was present. The patient’s medical record was then carefully reviewed. According to her chart, no epidural were ever placed at our institution. Preoperative available x-rays were also analyzed. Focusing on the area where the metallic image was expected, we surprisingly discovered it was visible in every exam including the interested region (Fig. 1). At further questioning, the patient recalled an epidural catheter being placed twelve years before, at an outside hospital, during the last trimester of pregnancy. Apparently, at the time, the device was placed to provide analgesia for a painful-rib-syndrome [4]. Particularly, she was experiencing excruciating pain in the upper abdomen-lower chest region, both anteriorly and posteriorly. As the patient’s pain was unresponsive to oral analgesics, the epidural catheter was placed (hence the low-thoracic location), and several cycles of analgesia were provided for about a month prior to labor through an elastomeric pump. The patient described the intervention as beneficial. Interestingly, she also recalled the catheter to be subcutaneously tunneled. After delivery, the pain immediately disappeared, thereby suggesting an association with pregnancy-related changes of the musculoskeletal structure. The epidural was removed in the immediate postpartum.

After discussion of the case between the anesthesia and surgical teams, including neurosurgical consultation, and considering the will of the patient, the patient was scheduled for urgent surgical removal of the fragment.

In the operating room, inspecting the skin of the right paravertebral region, a small scar could be observed, with a fibrotic reaction attributable to a foreign body lying just underneath. A looped radio-opaque object with its tip at the T12 level was then seen on intraoperative fluoroscopy. Examining lateral projections, showing most of the catheter to be superficially located, the attending neurosurgeon excluded the need for laminectomy. Under sterile conditions and with the patient in the prone position, local anesthesia was administered and incision of the skin and fascia was performed at the level of the scar. The proximal end of the catheter fragment was isolated about 1 cm below the incision and then carefully removed by slow constant traction.

**Fig. 2:**
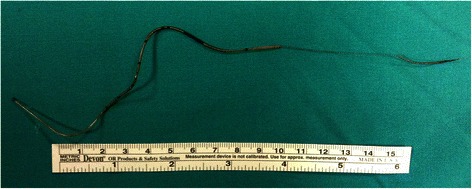
The foreign body. The sheared epidural catheter as surgically extracted from the patient. A terminal fragment of about 15 cm was retrieved, with a large uncoiled portion

A 15 cm fragment of a Flextip Plus 19G wire-reinforced epidural catheter (Arrow-Teleflex, Limerick, PA) was retrieved, with a large uncoiled portion close to its proximal end (Fig. 2). The operator verified the black terminal marker indicating tip integrity was present. Surgery was performed without any complication. A fluoroscopic control showed the absence of any metallic residual foreign body, and direct visualization during dissection of the skin confirmed that no portion of the uncoiled portion was left behind.

## Discussion

Shearing of an epidural is a rare but serious adverse event, whose management may be problematic for the anesthesiologist [5].

The greatest attention must be paid while handling the catheter, especially at its removal [6]. The fact that our fragment was retrieved with an already uncoiled large portion, suggests that the catheter broke during withdrawal, probably due to the operator inappropriately pulling the catheter through the subcutaneous tunnel. Generally speaking, careful training of the healthcare personnel is probably the most effective preventive strategy [7]. In addition, several technical options have been reported about what to do in case resistance is found during withdrawal of the catheter. Recommendations include (1) the use of slow continuous traction [8]; (2) interrupting the maneuver if the catheter begins to stretch, then reapply traction a few hours later [9]; (3) placing the patient in the lateral decubitus position, or the same position as insertion [6]; (4) extreme back flexion or extension [10]; and (5) the injection of a bolus of normal saline through the catheter [11].

For several reasons, our patient’s conditions demanded further meticulousness. First of all, the epidural was placed for an unusual pain management indication in a patient during her late pregnancy. The catheter was then removed postpartum. This must lead the operator to consider the physiological musculoskeletal changes of the vertebral column occurring after delivery, plus the possibility of epidural catheter migration [12]. Second, according to the patient, the catheter had been left in place for about a month. This should trigger further alarm, since a long-term implanted epidural elicits a granulomatous healing response by biological tissues. Third, it must be considered that the epidural was placed at a thoracic level. Compared to the lumbar region, the thoracic vertebrae offer narrower inter-laminar spaces and more acutely angulated spinous processes. This usually leads to a longer subcutaneous route of the catheter from the puncture site to the epidural space, thereby increasing the risk of entrapment [13]. Finally, our patient’s epidural was subcutaneously tunneled. Even if tunneling has its advantages, especially with long-term devices, attention must be paid at removal since the resistance to traction is likely to be greater than standard non-tunneled catheters [14]. Moreover, the tunneling process itself, if performed using the Tuohy needle as a guide, carries an increased risk of catheter damage [15].

As reported, in our case the retained fragment was repeatedly missed on plain X-rays and CT scans studies obtained during the patient’s 3-years history of recurrent diverticulitis. As much as it may sound surprising, it is actually well known that even the most expert observers often miss the occurrence of an unexpected- yet salient- event if they are engaged in a different task, a phenomenon called inattentional blindness [16]. It is likely that the off-focus metallic filiform object went unnoticed due to the troublesome history of the patient’s diverticular disease. In this case the clinician’s attention was directed to the identification of potentially life–threatening complications, such as abscess formation, bleeding, or perforation. Plus, a retained epidural tip by itself can be difficult to visualize through radiological imaging [17].

Interestingly, the fragment we retrieved came from a 19G Flextip Plus wire-reinforced epidural catheter. This device is made of polyurethane equipped with a stainless steel coil. The aim is that of providing a balance between robustness and flexibility. Whereas advantages have been shown regarding a reduced incidence of catheter migration, paresthesia, and accidental venous cannulation, a few reports in the literature can be found showing an increased tendency towards breakage of this specific kind of catheter compared to non-reinforced models [18].

For 12 years our patient unknowingly carried the fragmented epidural within her body. Therefore this case seems to indirectly support the approach according to which an asymptomatic fractured epidural can be left in place, being sterile and inert, with no urgent surgical removal demanded [5]. As described, in this particular situation, the foreign body was removed because of (1) its fairly large size; (2) the mainly subcutaneous location; (3) the evidence of a granulomatous scar suggesting a superficial location of the most proximal end; (4) the will of the patient. It is likely that no complications would have ever occurred if a conservative approach had been pursued. However, we firmly believe that the fact of unknowingly carrying a metallic foreign body bears its considerable risks. First of all, like in our case, the incidental visualization of the fragment at imaging obtained for different purposes might mislead the evaluation of conditions regarding the same anatomical district. Our patient underwent two major surgical interventions in a short time lapse, the second of which was urgent in nature. The visualization of such metallic filiform object at a postoperative X-ray prompts the exclusion of a retained surgical item. Second, given that the broken epidural catheter was missed, it is conceivable that more serious complications such as hematoma or infection may have also been discovered with a significant delay. Finally, the fragment might have caused potentially serious problems in instances such as the placement of a new epidural catheter, or the excessive heating that might result from exposure to strong magnetic fields like those of magnetic resonance imaging [19]. The only limitation of this case report is that, at the time of publication, the patient has not requested any documentation from the outside hospital, and our information from that hospitalization is solely based on her account of the events. Therefore, neither we can thoroughly comment on the original insertion and removal techniques, nor on any possible record of the epidural breakage event being present.

## Conclusion

This report describes the case of a patient who unknowingly carried an asymptomatic broken epidural catheter within her paravertebral structures for 12 years. After being repeatedly missed, the 15 cm fragment was finally identified at an abdominal x-ray obtained for an unrelated medical condition, of which it initially confounded the follow-up. The foreign body was then surgically removed.

We believe the case of this particular patient to be of great educational value. The long-term retention of the sheared fragment and its repeatedly missed identification, are representative of how this complication can go undetected, thereby exposing patients to preventable risk.

Adequate training of every health care provider potentially involved in removing epidurals is of utmost importance in terms of prevention. In case breakage of the catheter occurs, full disclosure and timely communication to the patient are essential, as well as detailed reporting in the hospital record. We recommend the statement “tip of epidural catheter intact” to be included in the patient’s chart upon uneventful removal.

To our knowledge, there is no report in the literature of such a large fragment being retained for more than a decade before being incidentally identified. Due to the rarity of this event and the lack of consensus on how it should be handled, this report adds to the currently available amount of evidence about this known complication and its management.

### Consent

Written informed consent was obtained from the patient for publication of this case report and any accompanying images. A copy of the written consent is available for review by the Editor of this journal.

## Abbreviations

CT: Computed Tomography
